# Supplementary material for: Data fusion and multivariate analysis for food authenticity analysis
Source: Nat Commun. 2023 Jun 8;14:3309. doi: 10.1038/s41467-023-38382-z (PMC10250487; doi:10.1038/s41467-023-38382-z)
Supplement: Supplementary file 1 — Supplementary Information [file 41467_2023_38382_MOESM1_ESM.pdf]

Supplementary Information

Data Fusion and Multivariate Analysis for Food Authenticity Analysis

Yunhe Hong[1], Nicholas Birse[1], Brian Quinn[1], Yicong Li[1], Wenyang Jia[1], Philip McCarron[1], Di Wu[1], Gonçalo Rosas da Silva[1], Lynn Vanhaecke[1,2], Saskia van Ruth[3,4] and Christopher T. Elliott[1,5]

1) National Measurement Laboratory: Centre of Excellence in Agriculture and Food Integrity, Institute for Global Food Security, School of Biological Sciences, Queen's University Belfast, United Kingdom
2) Laboratory of Integrative Metabolomics, Department of Translational Physiology, Infectiology and Public Health, Faculty of Veterinary Medicine, Ghent University, Merelbeke, Belgium
3) Food Quality and Design Group, Wageningen University and Research, Wageningen, Netherlands
4) School of Agriculture and Food Science, University College Dublin, Dublin 4, Ireland
5) School of Food Science and Technology, Faculty of Science and Technology, Thammasat University, 99 Mhu 18, Pahonyothin Road, Khong Luang, Pathum Thani 12120, Thailand

Corresponding Author

Christopher T. Elliott: chris.elliott@qub.ac.uk

Figure S1. Mass spectra. **a** Alaskan salmon, **b** Norwegian salmon, **c** Scottish salmon, **d** Icelandic farmed salmon, and **e** Icelandic wild salmon samples with REIMS

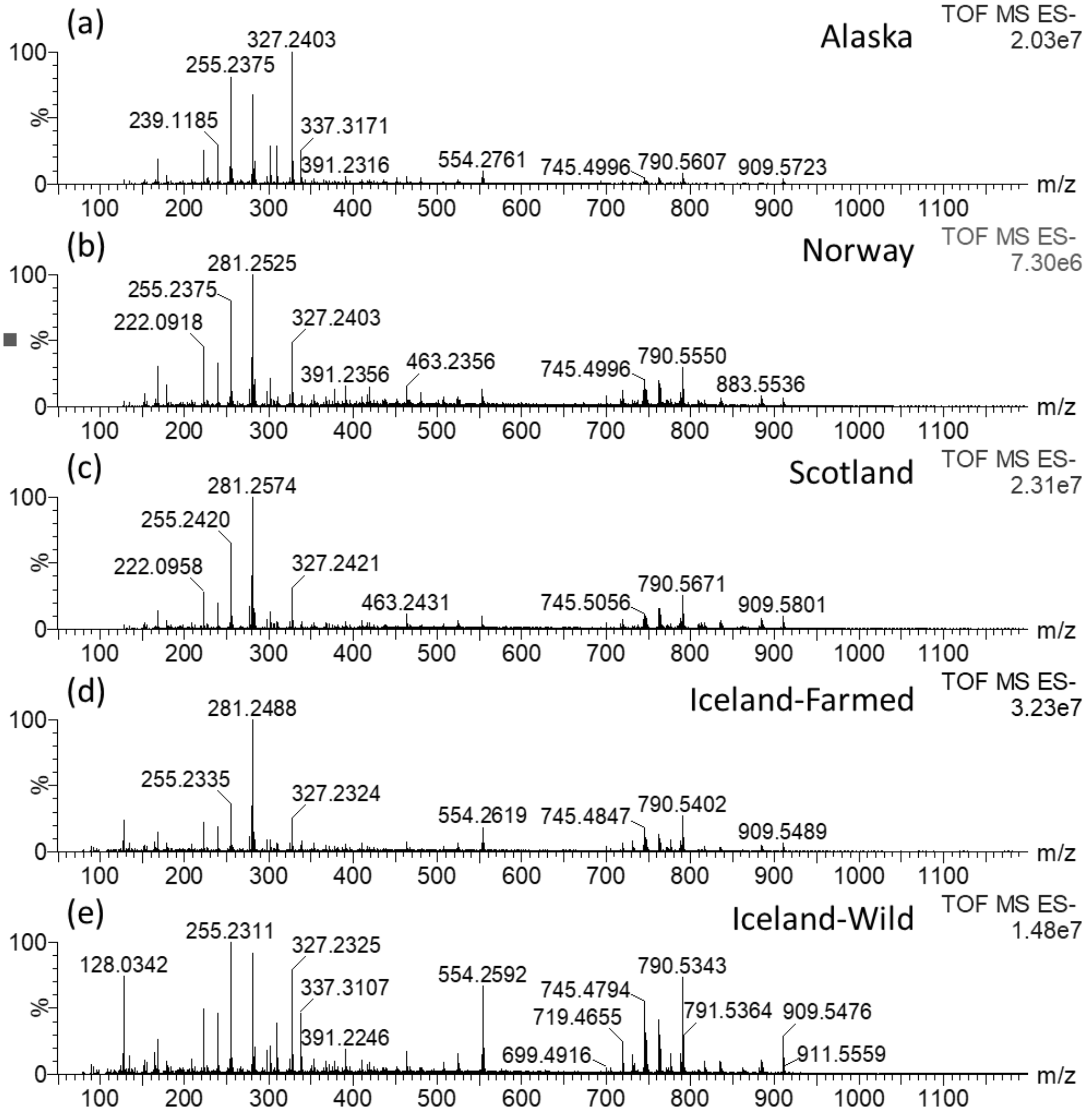

Figure S2. OPLS-DA between Icelandic farmed and wild salmon and Permutations plot of OPLS-DA model.

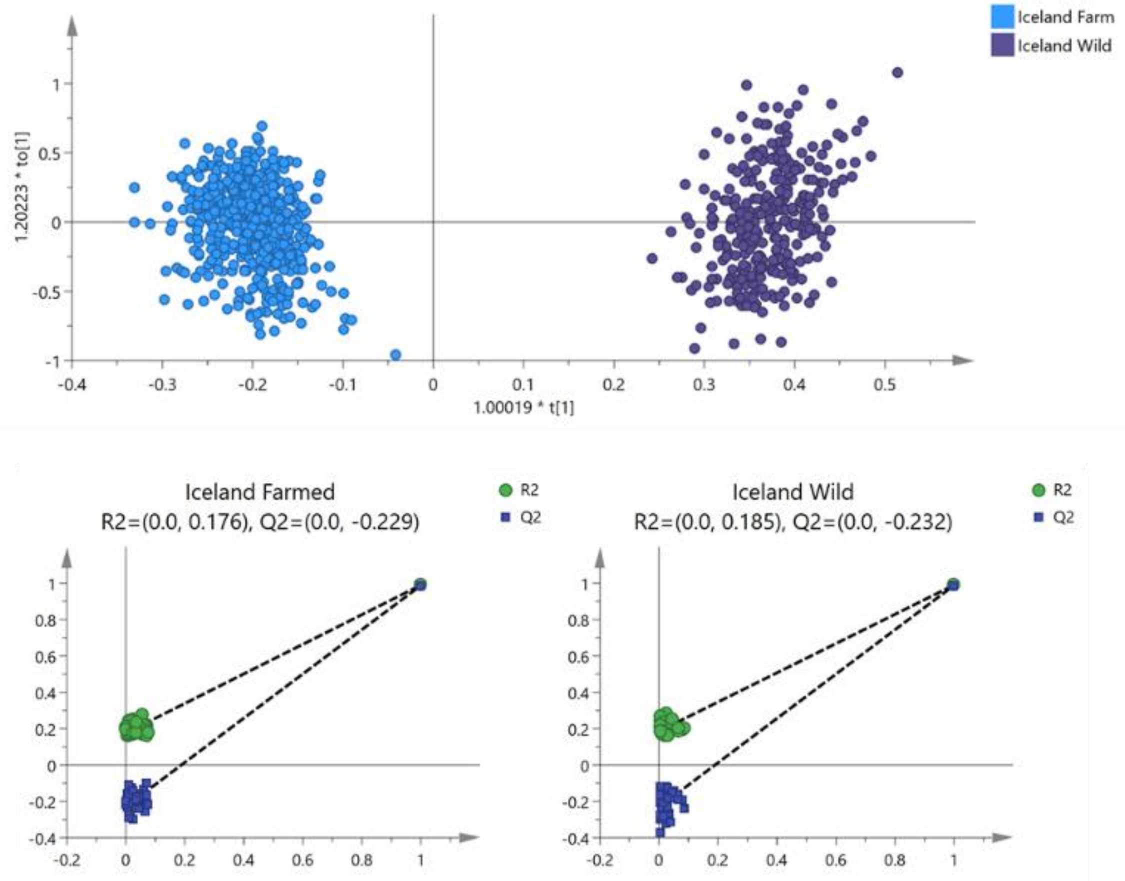

Figure S3. Candidate lipid biomarkers marked in OPLS-DA/S-plot of ions responsible for salmon geographical origin classification.

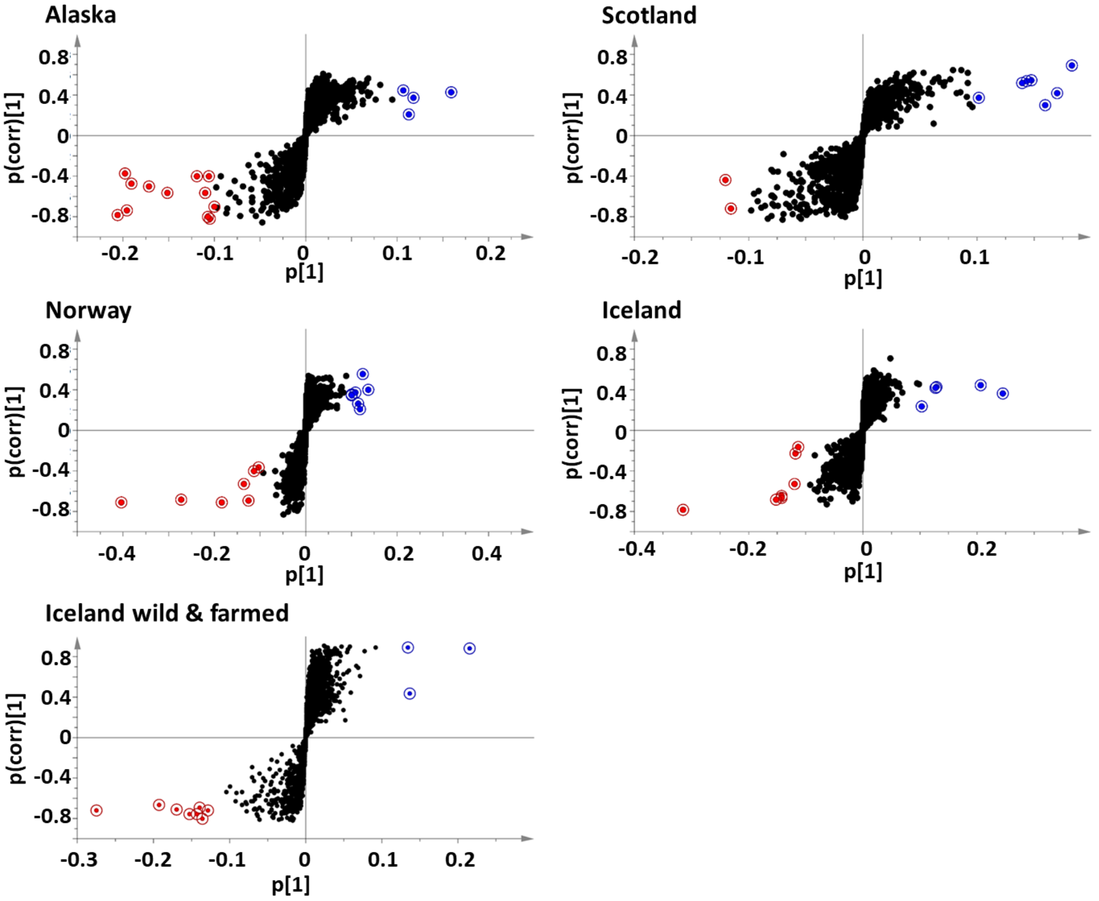

The variable contributions and sample correlations are represented on the X- and Y-axes, respectively. The further a data point is from 0, the more it contributes to sample variance. Data points with a difference of more than 0.1 were considered differentiators and labelled as biomarkers between any two groups.

Figure S4. Supervised learning models for differentiation salmon geographical origin. **a** LDA score plot of LD1 and LD2 amongst 5 salmon groups. **b** PLS-DA score plot of P1 and P2 amongst 5 salmon groups. **c** OPLS-DA score plot of P1 and P2 amongst 5 salmon groups.

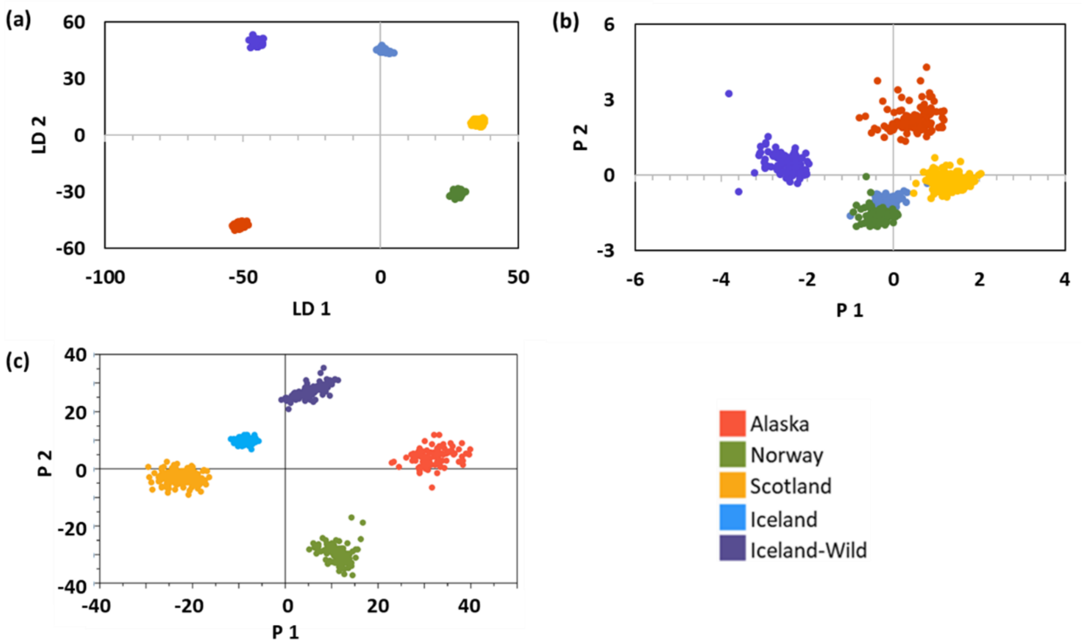

Figure S5. Validation of the salmon geographical origin models using the prototype recognition software and a further set of salmon samples purchased from the UK supermarket.

a The sample under investigation origin from Alaska and the figure above demonstrates the recognition software correctly identifying a sample burn to be Alaska (red circle, 6 replicants of each sample).

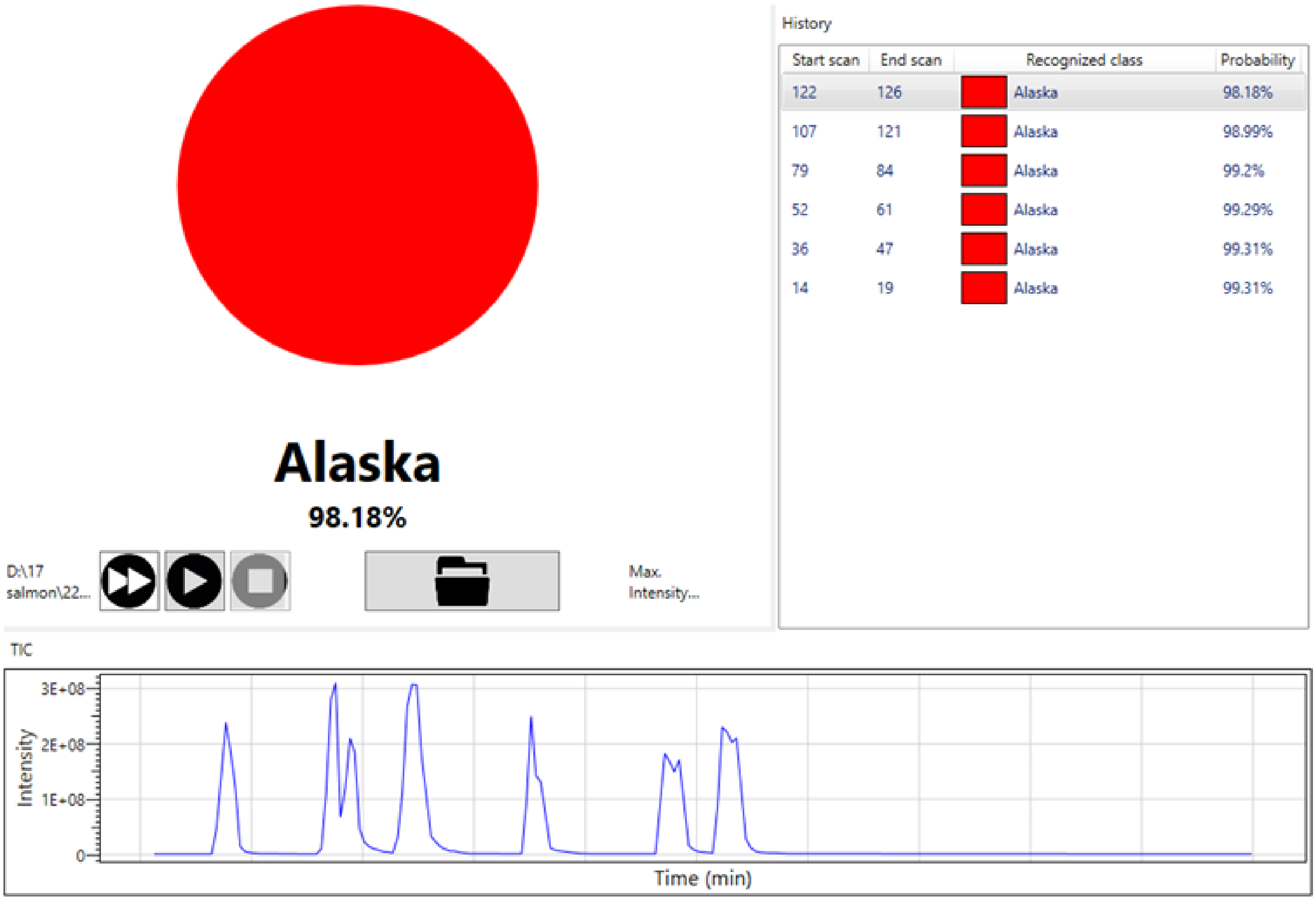

Figure S5. b The sample under investigation origin from Scotland and the figure above demonstrates the recognition software correctly identifying a sample burn to be Scotland (blue circle, 6 replicants of each sample, but only recognized 5 of these replicants).

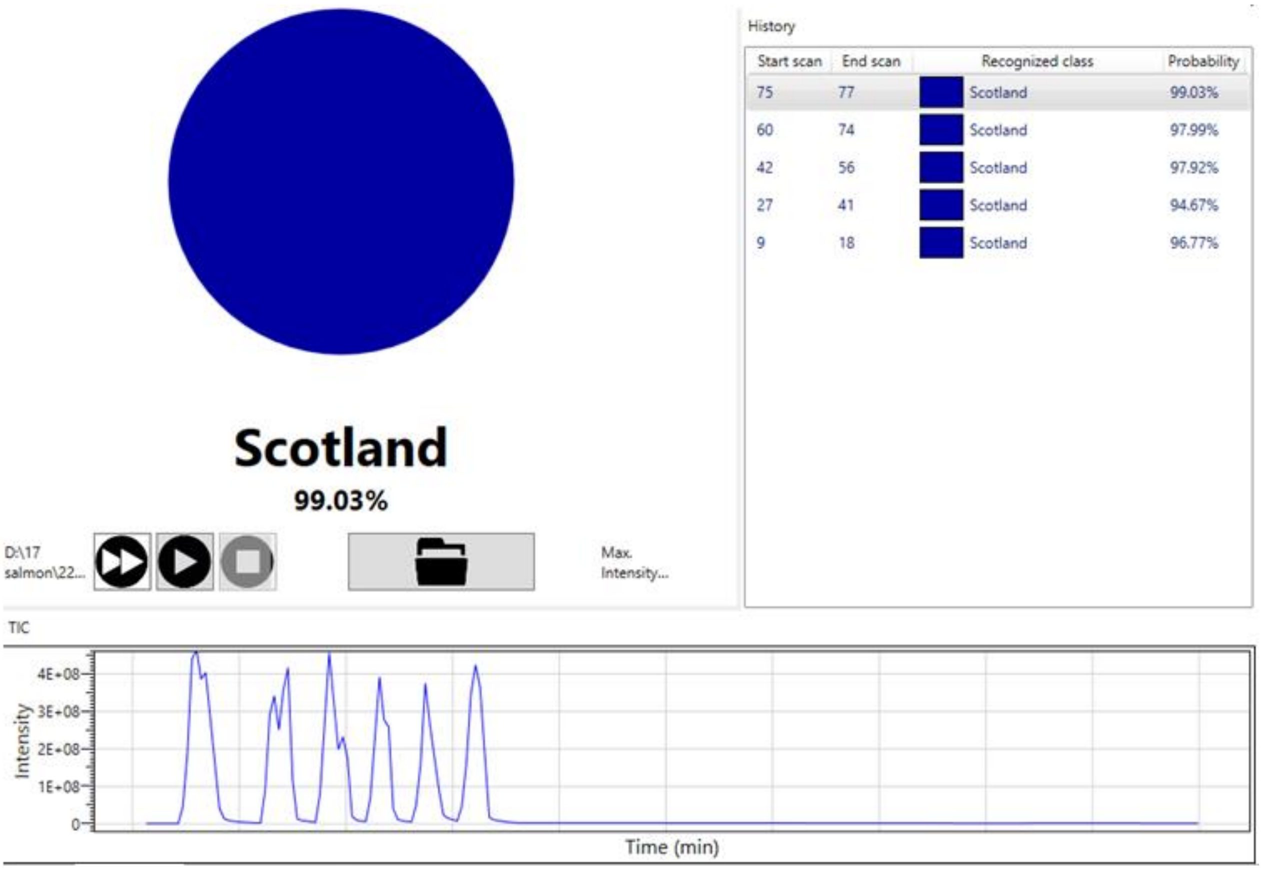

Figure S5. c The sample under investigation origin from Scotland and the figure above demonstrates the recognition software correctly identifying a sample burn to be Scotland (blue circle, 6 replicants of each sample, but only recognized 1 of these replicants).

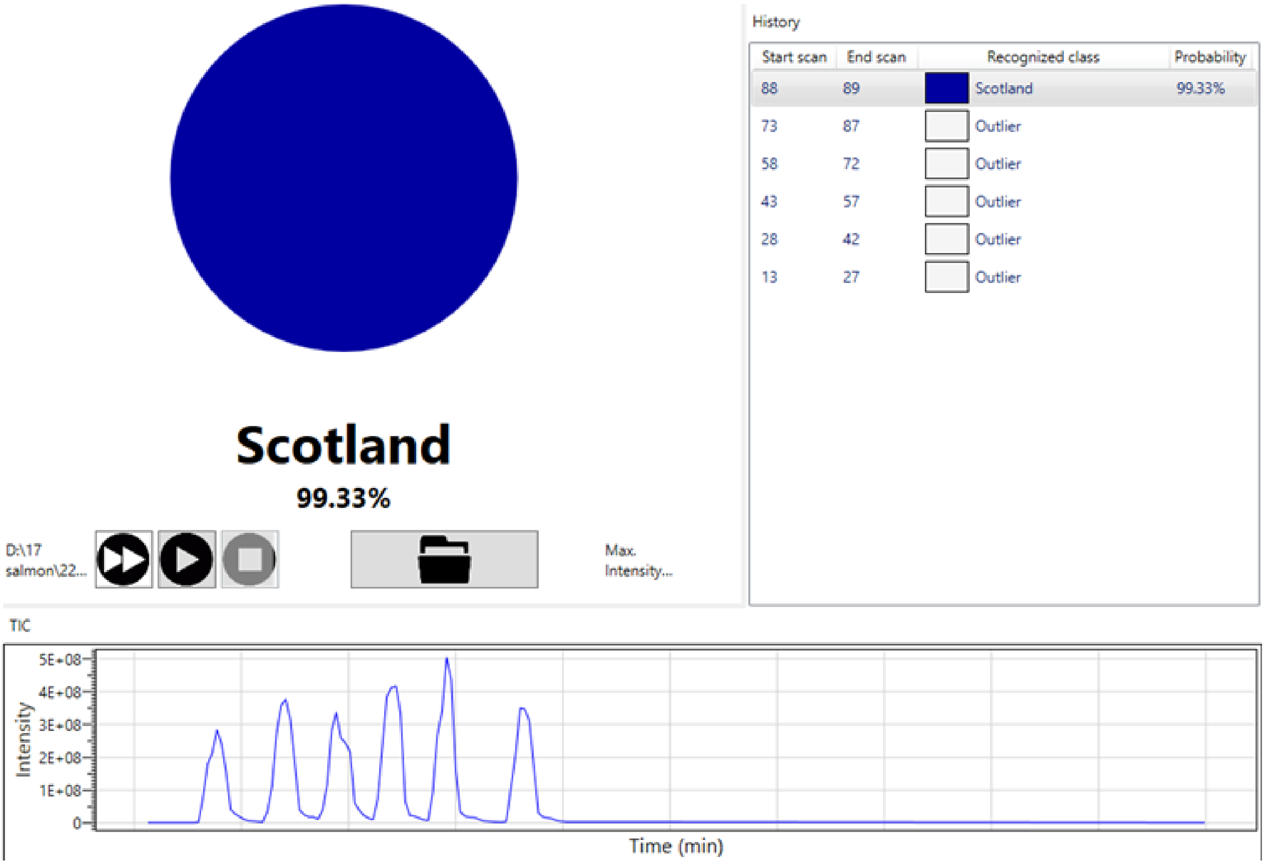

Figure S6. REIMS classification results by using 18 biomarkers. a PCA plot using 18 biomarkers. **b** LDA plot using 18 biomarkers. **c** test samples (n = 17, 6 replicants of each test sample) identification table using LDA model. Correct (%), percentage of correctly classified labels for salmon origins. Rows: labels, columns: predicted labels.

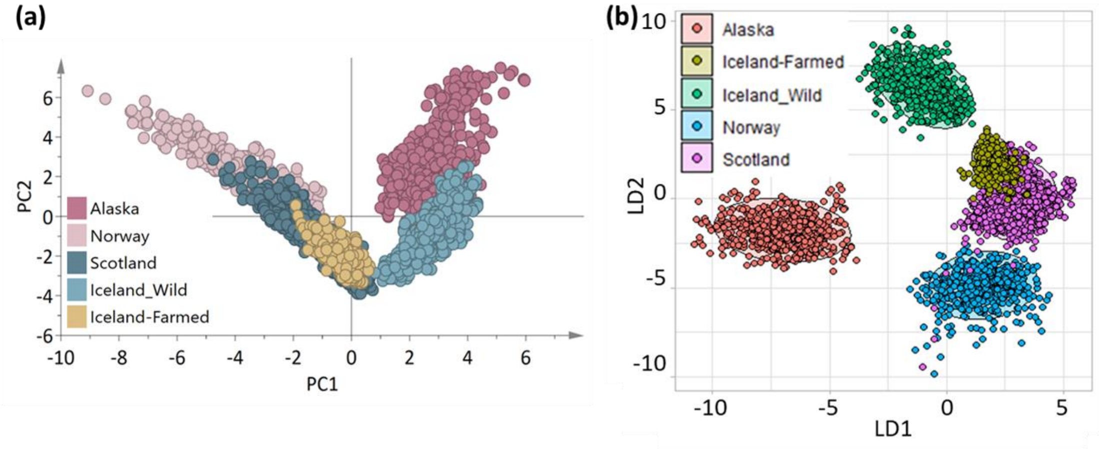

(c)

	Members	Alaska	Norway	Scotland	Iceland-F	Iceland-W	Correct
**Alaska**	42	31	0	11	0	0	73.8%
**Norway**	0	0	0	0	0	0	—
**Scotland**	60	0	33	27	0	0	45.0%
**Iceland-Farmed**	0	0	0	0	0	0	—
**Iceland-Wild**	0	0	0	0	0	0	—
**Total**	102	31	33	38	0	0	56.8%

Figure S7. ICP-MS classification results by using 9 elements markers. a PCA plot using 9 markers. **b** OPLS-DA plot using 9 markers. **c** test samples (n = 17, 6 replicants of each test sample) identification table using OPLS-DA model, Correct-percentage of correctly classified labels for salmon origins, Rows: labels, columns: predicted labels.

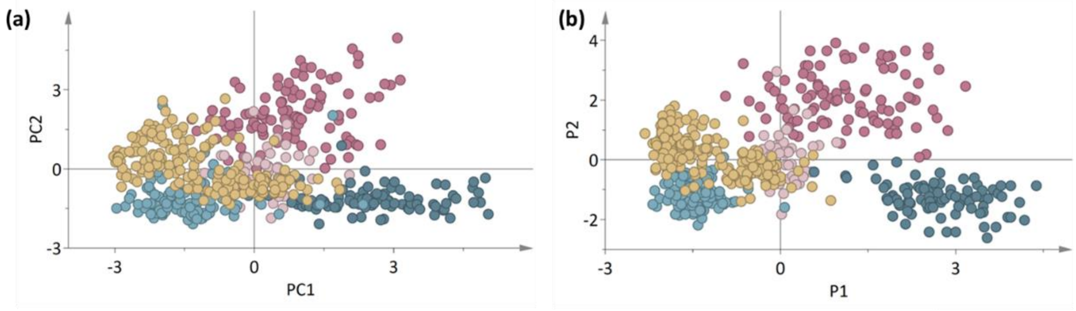

(c)

	Members	Alaska	Norway	Scotland	Iceland-F	Iceland-W	Correct
**Alaska**	42	18	12	7	0	5	42.9%
**Norway**	0	0	0	0	0	0	—
**Scotland**	60	0	42	10	6	2	16.7%
**Iceland-Farmed**	0	0	0	0	0	0	—
**Iceland-Wild**	0	0	0	0	0	0	—
**Total**	102	18	54	17	6	7	56.8%

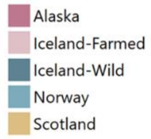

Table S1. Statistics table of biomarker distribution in five salmon groups.

Mass Bin	Alaska	Scotland	Norway	Iceland	Iceland-Wild	Count
127.1				✓	✓	2
128.1	✓		✓	✓	✓	4
222.1		✓	✓			2
239.1	✓	✓	✓			3
255.3	✓			✓		2
277.3	✓		✓		✓	3
279.3	✓		✓		✓	3
280.3			✓		✓	2
281.3	✓		✓		✓	3
282.3			✓		✓	2
301.3	✓	✓				2
309.3	✓	✓				2
327.3	✓	✓	✓	✓	✓	5
337.3	✓	✓	✓		✓	4
338.3	✓					1
745.5		✓	✓			2
790.5			✓		✓	2
909.5			✓			1

Table S2. PCA-LDA cross-validation table for salmon origin identification included in the test samples (n = 17, 6 replicants of each test sample). Correct (%), percentage of correctly classified labels for salmon origins. Rows: labels, columns: predicted labels. (REIMS)

	Members	Alaska	Norway	Scotland	Iceland-F	Iceland-W	Outlier	Correct
Alaska	99	99	0	0	0	0	0	100%
Norway	100	0	100	0	0	0	0	100%
Scotland	183	0	0	183	0	0	0	100%
Iceland-Farmed	50	0	0	0	50	0	0	100%
Iceland-Wild	90	0	0	0	0	90	0	100%
Total	522	99	100	183	50	90	0	100%
Test samples	102	42	0	42	0	0	18	82.3%

Table S3. Model correct classification rate comparation results using five-fold cross-validation and the 17 test samples (6 replicants of each test sample) identification results. Rows: labels, columns: predicted labels. (ICP-MS)

	**Members**	**Alaska**	**Norway**	**Scotland**	**Iceland-F**	**Iceland-W**	**Outlier**	**Correct**
**Alaska**	**99**	**96**	**0**	**1**	**0**	**2**	**0**	**97.0%**
**Norway**	**100**	**1**	**96**	**3**	**0**	**0**	**0**	**96.0%**
**Scotland**	**183**	**0**	**1**	**182**	**0**	**0**	**0**	**99.5%**
**Iceland-Farmed**	**50**	**0**	**0**	**8**	**42**	**0**	**0**	**84.0%**
**Iceland-Wild**	**90**	**0**	**0**	**0**	**0**	**90**	**0**	**100%**
**Total**	**522**	**97**	**97**	**194**	**42**	**92**	**0**	**100%**
**Test samples**	**102**	**7**	**16**	**78**	**1**	**0**	**0**	**64.7%**

Table S4. 17 test salmon samples ID and the origin authenticity identification results by using REIMS, ICP-MS, and data fusion coupled to machine learning respectively (6 replicants of each sample).

**Sample ID**	**Label Origin**	**Sample analysis results**		
		**REIMS**	**ICP-MS**	**Data Fusion Coupled to Multivariate Analysis**
WA001	**Alaska**	Alaska	Alaska/Iceland wild	Alaska
WA002	**Alaska**	Alaska	Alaska	Alaska
WA003	**Scotland**	Outlier	Scotland	Scotland
WA004	**Scotland**	Scotland	Scotland	Scotland
SA001	**Scotland**	Scotland	Scotland	Scotland
SA002	**Scotland**	Scotland	Scotland	Scotland
SA003	**Alaska**	Alaska	Iceland farmed/Norway	Alaska
SA004	**Alaska**	Alaska	Norway/Scotland	Alaska
MS001	**Scotland**	Scotland	Scotland	Scotland
MS002	**Alaska**	Alaska	Norway	Alaska
AL001	**Scotland**	Outlier	Scotland	Scotland
LD001	**Scotland**	Scotland	Scotland	Scotland
MR001	**Scotland**	Scotland	Scotland	Scotland
TE001	**Alaska**	Alaska	Norway	Alaska
TE002	**Alaska**	Alaska	Scotland	Alaska
TE003	**Scotland**	Outlier	Scotland	Scotland
AS001	**Norway and/or Scotland**	Scotland	Scotland	Scotland
